# Mr. Christie's Observations on Gun-Shot Wounds

**Published:** 1800-02

**Authors:** 


### *Mr. Christie's Observations on Gun-shot Wounds.*

[ Continued from our last Number, p. 40. ]

THE general confusion which prevails during an action, will amply account for wounds being less regularly dressed, or operations less nicely performed, by the regimental practitioner in the field, than in the general hospitals. In short, it can hardly be done, and is therefore seldom expected. I believe the duties of the regimental medical department have been, in general, as faithfully discharged, during this part of the campaign, as trying circumstances, destitute of many resources, could possibly admit of, and particularly in the action at the village of Oude-Caspel, on the 19th of September; when the Guards, the 9th, 17th, 35th, and 40th, English regiments, along with the Russians, maintained so obstinately, so severely, their disputed ground; I heard it (though at a distance) reiterated by many a grateful tongue, the handsome, the heroic manner in which several of the regimental medical staff discharged their soothing duty on the scene of action, aided by few means, and when all Nature seemed to be waging war with the healing art; even then lives were saved by judicious and timely benevolence, volence. I have taken the liberty of mentioning this, becaufe a fpirit of bravery, wherever it appears neceffarily adorned, as it muft be, by many virtues, ought not to be paffed over unnoticed.

In the application of bandages, along with compreffes varioufly modified as it can be, the roller will be found to anfwer for any part of the body, where the wound is flefhy. In the limbs, where there is fracture, the eighteen or twelve-tailed bandage ought invariably to be ufed; and as the application of fome emollient poultice becomes neceffary to foothe the inflammatory action, which foon commences after every wound, this fort of bandage is much more handy than any other, and ought perhaps to be more frequently ufed than it is at prefent. Of late it has been very cuftomary to place fractured limbs in an eafy pofition on a pillow, covering them over merely by the fracture cradle; the cure is thus rendered comfortable; but it is plain, cannot be adopted in moving armies, or on fhipboard.

For inflamed parts we meet with two kinds of poultice, oppofite in their nature, recommended by writers, namely, the faturnine and emollient. The preparations of lead in poultice, as well as in wafh, for the repulfion of inflammation, are much in ufe in Scotland, chiefly from its being recommended by a defervedly eminent and refpected writer of that country. It requires, however, a nice care and confiderable experience in the proper ufe of this fort of poultice. When the tenfion is great, the inflammation running high, and advancing rapidly, this is very apt to do mifchief; while, on the other hand, the emollient poultices always foothe, and alfo, by their relaxing quality, often repel, even when applied with a view of haftening fuppuration. It is after proper evacuations in the incipient ftage of inflammation, and in the more indolent kinds, that I have found the applications of lead ufeful in effecting refolution. In gun-fhot wounds it is probable they ought never to be had recourfe to, for they will certainly tend to increafe tenfion. The common bread and milk poultice with oil, and the linfeed poultice, are found to anfwer every purpofe. A very convenient one, when thofe cannot be had, as will frequently happen to the military practitioner, is made from a maceration of oatmeal in water, which takes up the glutinous and finer parts, leaving behind the groffer particles. The infufion is to be boiled to a proper confiftence, and applied with or without oil. This method I had from a furgeon in the navy, where this kind of poultice, I underftand, is very common and perfectly convenient.

In foul and floughing fores, the fermenting poultice, as one made of yeaft and meal, will be found preferable. In thofe kinds kinds of sores, too, the carrot and turnip poultice have been much recommended; and it is said they all do good by the fixed air (carbonic acid gas) which they contain; with the same view, no doubt, has the charcoal poultice been used.

With respect to the extraction of balls, and other foreign matter, it is now, I believe, a rule not to attempt it, unless when readily within our reach, or placed in a situation that gives intolerable irritation; or where life might be endangered by its remaining. When out of reach, the ball will easily come out by the suppurative process which follows; and the many instances on record of balls remaining for life, forming for themselves an harmless bed, or working themselves out to other parts of the body, are numerous, and need not therefore be farther noticed here.

In gun-shot wounds it rarely fails that a considerable degree of febrile action commences, and I know no instance wherein the free use of the lancet affords greater relief; though the intestines are also to be kept open, purging, for obvious reasons, cannot well be fully employed. Opiates, in full doses, must also occasionally be had recourse to.

Of the easiest and most effectual method of preventing fistulous sores (which we might at first suppose frequently, but which in fact are seldom found) following those wounds, I have not yet had sufficient opportunities to determine.

Having thus, Gentlemen, completed my design, in giving you my introductory paper on gun-shot accidents, I will hardly think of forming an apology for the hasty and inaccurate manner in which it has been sent you. The duties of the field, admit of neither system nor elegance;—utility has been my constant aim;—how far in my papers I may be likely to succeed, it is for you to judge; but my labours in this service have been occasionally repaid by gratitude, a recompense I am proud to acknowledge, and which shall never be effaced.

---

### *To the Editors of the Medical and Physical Journal.*

Gentlemen,

In the observations on gun-shot wounds, which I transmitted from Holland, and which you, did me the honour of inserting in your Journal; I noticed the serpentine course which a wound from musket shot so frequently assumes; I mentioned this not merely as a matter of curiosity, but to shew, that that uſeful practical facts would ariſe from the remark. Having lately paid ſome attention to a variety of caſes, I find, that in almoſt every gun-ſhot wound, the courſe of the ball had been more or leſs ſerpentine; for a body moving obliquely, has its courſe inſtantly altered, on *touching* a denſer medium; as may be ſhown from the riſing and falling of cannon or ſmall ſhot, when fired along the ſurface of the ſea. Now, when we conſider the ſkin, muſcles, blood veſſels, membranes, and bone, as ſo many different media, (and from their difference in denſity, they in fact are ſo) we ſhall, perhaps, ſcarcely find one gun-ſhot wound whoſe courſe can be ſaid to be truly ſtraight. The ball meeting with different media variouſly placed, its courſe will be neceſſarily varied.

In my paper, I was anxious to allege, that the velocity and turning courſe by which balls enter the cavities of the body, might tend to prevent miſchief, by their thus excluding the external air. I am perfectly aware, how many profeſſional men and teachers, celebrated too for acuteneſs of diſcernment, have ſcoffed at this obſervation; but with all deference to opinions, I am yet ſatisfied, that the acceſs of the external air, is, in many inſtances, ſufficient to account for the dreadful conſequences that ſo frequently enſue it's entrance into ſacs naturally ſhut. Mr. John Bell, whoſe eminence as a ſurgeon is acknowledged, whoſe acuteneſs is well known, and whoſe talent for inſtruction I have often admired, doubts that the external air applied to ſhut cavities, ever produces injurious conſequences. "I do affirm," ſays he,* "that it remains to be proved, that this fluid, which ſeems ſo bland and pleaſant to all our ſenſes, and to the *outward ſurface*, is yet a horrible ſtimulus, when admitted into ſhut cavities." To this remark, I would beg to reply, that an ounce of Port wine thrown into the abdomen or ſcrotum, will likely excite inflammation in thoſe parts, while a bottle of the ſame fluid taken into the ſtomach, will only produce a pleaſant emotion. Mr. Bell inſtances too the caſes of hernia, and other protuſions of inteſtine not followed by inflammation, as a proof of the mildneſs of air. It will hardly be denied, however, that the peritoneo-inteſtinal inflammation, which ſometimes ſucceeds after the operation for hernia, is moſt probably aſcribable to this expoſure; from analogy we may be allowed to ſuppoſe, that much will depend on the length of time of expoſure, for we know that a ſtimulating injection thrown into the cavity of the tunica vaginalis teſtis, will ſometimes fail in bringing on inflammation from the fluid

* Diſcourſes on Wounds.

fluid, not being applied for a ſufficient length of time. The inſtance which Mr. Bell adduces to corroborate the correctneſs of his doctrine, that the air effuſed in emphyſema does not produce inflammation, I think, is an argument not at all admiſſable; for it muſt be recollected, that emphyſema is occaſioned almoſt only by a wound which penetrates the pleura and ſubſtance of the lung, without an external opening, ariſing from a fractured rib: now, the air which eſcapes into the cellular ſubſtance muſt, in this caſe, iſſue from a ſmall opening only, previous to which it muſt have undergone the action of the lung, and thus be deprived of its chief ſtimulating principle, namely, the oxygen.

A celebrated profeſſor,* whoſe obſervations are no leſs remarkable for their ingenuity than juſtice, long ſince taught, that a radical cure for hydrocele might be rationally attempted, by throwing air into the cavity of the tunica vaginalis teſtis, after the evacuation of the water; and there is little doubt but his idea ſuggeſted the operation which, I believe, is now generally uſed for this diſeaſe, namely, the ſtimulating injection of wine or other fluids.

Of the proximate cauſe of inflammation generally, but particularly that following the *puncture* of ſhut cavities and their contents, there appears ſomething difficult to be explained. A prick with a bayonet or ſtiletto, is very frequently followed by fatal conſequences, while an alarming protruſion of the bowels, occaſioned by the cut of a ſword, ſhall be unattended with any bad ſymptom.

A regiment to which I was attached in Flanders in 1794, by miſtake in a dark night, while marching, had got cloſe upon the French poſts; in retiring, the prancing of ſome horſes in the rear, occaſioned a confuſion, which was communicated to the whole corps with the irreſiſtible rapidity of a torrent. The road was narrow, with a ditch on each ſide. One man had the humerus luxated by a fall; ſeveral were bruiſed by being trod upon; one man ran himſelf againſt a bayonet, which entered a little way into the left ſide, between the falſe ribs and ſpine of the ilium; a trifling hæmorrhage followed; a compreſs and bandage were applied over the wound, and he was largely blooded next day, with a view of preventing, as much as poſſible, any bad conſequence; two days afterwards, however, he was attacked with ſymptoms of peritoneal inflammation. The regiment was then conſtantly moving, without any conve-

* Monro.

nences; he was taken to a general hospital, where, in spite of bleeding and other means, he soon died.

A soldier of the same corps, who had been bred a lawyer, was reduced from being a serjeant, to serve in the ranks as a private, when quartered at Ealing in Hampshire, in 1796; he was determined to take the *law* in his own hands, for he attempted to stab himself with his pen knife; there were two small cuts in the abdomen, above the umbilicus, through which the omentum could be seen; it did not appear, however, to be divided. The wounds were closed, and kept so by sticking plaster; and, by way of preventative, he was largely bled more than once; no violent symptoms ever occurred, and he perfectly recovered.

A soldier in the same regiment, at Columbo, in the island of Ceylon, in 1797, had been carelessly cleaning his firelock, with the bayonet fixed, and placed over the foot of his bed. One of his comrades ran himself against it, entering somewhere below the umbilicus; he thought so little of the accident that he would hardly be prevailed upon to report to the attending mate; who, in his turn, declared it was trifling; and having put some superficial dressing over the wound, said, nothing bad would follow. In two days afterwards, however, the man began to be attacked with symptoms of peritoneo-intestinal inflammation, which destroyed him in four-and-twenty hours. Those cases occurred in healthy men, in whom the abdomen was completely filled with the viscera; there was, therefore, no possibility of the external air getting in to produce any harm. The case in which there was no division of parts within the parietes of the abdomen, recovered; the other two, in which there was a puncture in some part of the viscera, were followed by death. In the whitloe, the peculiar severity of the inflammation arises from its being seated in membraneous parts, and confined by unyielding substances. The old hypothesis of an error loci, or a globule of blood having got into a vessel, unequal to transmit it, and this producing an effort of the vis conservatrix naturæ,* appears

---

* This was part of the Boerhaavian system, in inflammation arising without any evident external cause. This doctrine also taught a lentor of the fluids, or part of th m, becoming præternaturally dense. One objection to this hypothesis has been, that the blood is supposed to be thinner in inflammation than in health; and as the buffy coat on the surface of the crassamentum is generally allowed to indicate the presence of inflammation, it has been received, that this appearance is produced merely by the red globules separating themselves from the coagulable lymph, which is thus shewn on the surface in its natural colour, but on account of its diseased thinness, is it appears to me, not more exceptionable than other hypothefes; for, in whitloe, it will hardly be denied, that the difordered action had arifen from a caufe feated in a minute point, although its confequence might fpread over the whole hand. Again, the practice among fome army furgeons (whom we may well fuppofe are oftener guided by experience than reafoning,) of fcarifying deep-feated gun-fhot wounds in flefhy parts, giving the blood vent, as it is called, and allowing *room* for the parts to fwell, feems to favour this opinion, namely, that in thofe bayonet wounds of the abdomen, the mifchief happens from membranous parts being principally concerned, which do not admit of fufficient yielding, and the confequent effort of Nature brings on general peritoneal inflammation. Thus, I imagine, it has happened, that a protruded portion of found inteftine has been returned into the abdomen without any bad confequence; and yet, had the flighteft puncture been made on that portion, it would, probably, have laid the foundation of a fatal peritoneo-inteftinal inflammation. In inflammation of the eye, when I thought I *frequently* gave relief by dividing a turgid branch or two of a veffel on the albuginea, I muft, at the fame time acknowledge, that *fometimes* the practice appeared to do harm; for, in a day or two afterwards, a vaft number of fmaller veffels, hitherto invifible, would fhew themfelves running out from the place that had been divided; now, were thofe blood veffels confined to a fubftance before, as unyielding as the fclerotica is behind them, I make no doubt that an inflammation of the moft violent kind would have enfued. But how miferably, alas! do we often fail when attempting to elucidate the operations of Nature! how frequently fhe eludes us when trying to fubjugate her under rude mechanical laws! To mark accurately, *effect*, I believe, will ever tend more to the improvement of the healing art, than by bewildering the imagination in the endlefs mazes of *caufe.* Why is it, that a bruife on the heel fhall, in one perfon, bring on a fatal tetanus, while another, of a fimilar habit, and in the fame climate, fhall have his leg compoundly broken, without any alarming phænomenon?

---

is incapable of fufpending the red globules as in health. Does not the greater contraction of the craffamentum in inflammatory difeafes, and the cupped appearance of its furface, lead us to imagine that the red globules may have acquired a truly præternatural denfity? It will, I hope, not be impertinent here to obferve, that inflammation may, and does, fometimes exift without the blood fhewing a buff-coloured appearance, efpecially in the firft drawing; and again, blood fometimes fhews this coat without there being any reafon for fuppofing inflammation prefent.

 I think

I think that wounds of the cheſt, at leaſt thoſe where one lung only is concerned, are much leſs apt to be followed by fatal conſequences than penetrating wounds in the abdomen. It cannot certainly be denied, that the lungs are leſs important to life than any of the abdominal viſcera; the uſe of one lung may, with little inconvenience, be temporarily diſpenſed with; but, beſides this, the yielding ſubſtance of the lung admits the inflammation ſpreading, and thus *humouring* as it were, the vis conſervatrix naturæ: to this add, when a fluid is ſecreted or formed during thoracic inflammation, it will generally meet with a readier exit here than it could poſſibly find in the abdomen. In thoſe wounds of the cheſt where the external air had entered, the lung muſt, of neceſſity, become collapſed; if the lung itſelf be wounded, the collapſe will be attended alſo with ſuch a contraction of the wound, as will be very favourable for its union; hence, what appears to be one of the moſt dreadful conſequences of a wounded lung, becomes, in fact, a guidance to recovery. The difficulty of breathing in thoſe wounds becomes, indeed, truly alarming, for the lung, in its collapſed ſtate, ſimilar to the fœtal lung, is capable of circulating but a comparatively ſmall proportion of blood; hence, from this cauſe, as well as the extenſive inflammation which will generally be found to follow this accident, the uſe of the lancet becomes doubly indicated.

Here, I beg to conclude thoſe obſervations on wounds of the cheſt, by the caſe of Doogen, of the 27th regiment, whom I ſtated in my paper from Schagen to have been wounded the 27th of Auguſt laſt, and which appeared in the number of your Journal for December; he lately joined the regiment in perfect health, but on account of the want of a proper uſe of his arm, he is about to be recommended for Chelſea. On examining his caſe more particularly than I could the day he was wounded, I find the ball had entered immediately before the right axilla, between the ſecond and third ribs. The wound has been quite healed theſe two months; he cannot extend his arm fully nor eaſily on account of the contraction of the divided pectoral muſcles. After the accident, he ſays, he continued to expectorate, more or leſs, of a bloody froth, for a fortnight; two pieces of bone had exfoliated from the injured rib. Occaſionally, he ſays, he ſtill expectorates a little of a jelly-like matter ſtreaked with blood, without pain or any conſiderable exertion. Soon after the accident, he had been attacked with ſevere ſymptoms of pneumonia, ſuffered violent pain of his breaſt, frequent teazing cough, and very great difficulty of breathing, and by his own account was blooded largely twelve times in the courſe of ten or eleven days. At preſent, his appearance is healthy, and and even florid; his pulse about 86, abundantly full and regular; he cannot lie with ease on the left side; when in that position, he has a sense of weight, and cutting, as he terms it; neither does he lie with ease on his back, but is free from all inconvenience when lying on the affected side. He says, there was a crackling sort of swelling around the wound, which appeared some time after the accident; the ball still remains in. Here is a case, where a ball lodged certainly within the chest, and apparently in the very substance of the lung, followed by extensive pneumonic symptoms, and succeeded by complete recovery. It's permanence, however, can hardly be trusted; for, should Nature not attempt a process to move it outwards, it will possibly lay the foundation for pulmonary consumption, on some slight exciting cause. In this, as well as several similar cases, which I have had permission to see at the Great Military Hospital here, under the direction of John Whitlocke, Esq. I have no doubt but the external air had insinuated itself through the wound, had occasioned collapse of the lung, and inflammation of the pleura. In Doogen's case, the difficulty of breathing, which was evident immediately after the shot, seems clearly to have been occasioned by the collapse of the lung; the hæmorrhage, which was poured out in abundance from the wound, and by the mouth,* had relieved *this* in some measure, until the inflammation of the pleura had again required blood-letting. Lastly, the wound filling up, and gradually excluding the external air, the absorbents taking up what had entered, the lung gradually resumed its function. That absorbents and arteries have, indeed, the power of taking up or depositing air in a disengaged state, as well as other fluids, is rendered sufficiently probable from cases of tympany, and from the bags of air found in the structure of several vegetables, particularly in the sea-weed, kali, the air in which must certainly be deposited by vessels, which again will have a corresponding set of absorbents to regulate the quantity in the bag.† The preceding observations will tend to shew how emphysema so seldom happens after gun-shot wounds of the chest. This diffusion

* When I stated this case, I mentioned there was no hæmorrhage by the mouth, the little time I was with him, arising no doubt from the state of an approaching syncope in which I found him; for, on minute examination, I found blood had been thrown up before I saw him.

† In those hysterical flatulencies which are so frequently astonishing, and sometimes so alarming, in weakly women, do the eructations of air arise merely from the nature of the food taken? from a vitiated or diminished state of the gastric liquors? or, can we suppose air secreted by the vessels opening into the intestinal canal? Most probably there is a combination of the whole.

diffufion of air, it is evident, can only well happen, as before-mentioned, where the pleura and fubftance of the lung are penetrated *without* an external opening; happening, of courfe, moft frequently from a fimple fracture of a rib, or at leaft where the fracture is rendered compound by an internal laceration, by a fplinter pufhed inwards directly, or elfe by the play of the lung by time abrading the pleura.

I formerly obferved, that after a fhot where the ball remains lodged, Nature feems difpofed to accommodate the foreign body; a fort of fac is formed for it, and it may remain harmlefs for life, even in the deeper-feated and perhaps more important parts. Although this does fometimes happen, yet it more frequently appears, that after the conftitution has remained for fome time peaceable and quiet, and feemingly on good terms with her foreign vifitor, fhe fhews anxiety, figns of uneafinefs; fhe awakens, as it were, to a fenfe of her duty; hence, balls find their way to the furface, fometimes indeed by their gravity, but more commonly by an abforbing procefs, formed for the purpofe of conducting them fafely out of her dominion. We therefore find fuch foreign bodies taken out near the furface, where, fome time before, no trace of them could be found. In cafes of abfcefs and folid tumours too, we are told, Nature always fhews a difpofition to bring the offending body to the fkin, although, perhaps, there may be greater refiftance on this quarter to encounter. It is faid, fhe cautioufly avoids abforbing and giving way on that fide where effential parts refide; a procefs in itfelf moft beautiful, and well calculated to call forth our admiration towards a fupreme, pervading, adminiftering Being, whofe power, alone, would appear to us capable of directing the varied functions of animated Nature, amid fuch nicety, and with fuch furprifing order! Mundi Anima Deus eft.

*Deal, January,* 1800.

